# 176. A Quality Improvement Endeavor to Improve Antibiotic Prescribing for Children with Acute Otitis Media in Emergency and Urgent Care Settings

**DOI:** 10.1093/ofid/ofae631.056

**Published:** 2025-01-29

**Authors:** Joana Dimo, Matthew J Weber, Meghan C Birkholz, Irina Topoz, Nicole M Poole

**Affiliations:** University of Colorado/Children's Hospital Colorado, Denver, Colorado; University of Colorado/Children's Hospital Colorado, Denver, Colorado; Children's Hospital Colorado, Clifton, Virginia; University of Colorado/Children's Hospital Colorado, Denver, Colorado; University of Colorado School of Medicine, Aurora, Colorado

## Abstract

**Background:**

Acute otitis media (AOM) self-resolves without antibiotics in 60-75% of cases. This study evaluates the impact of a quality improvement intervention on antibiotic prescribing for pediatric AOM.

**Figure 1.**
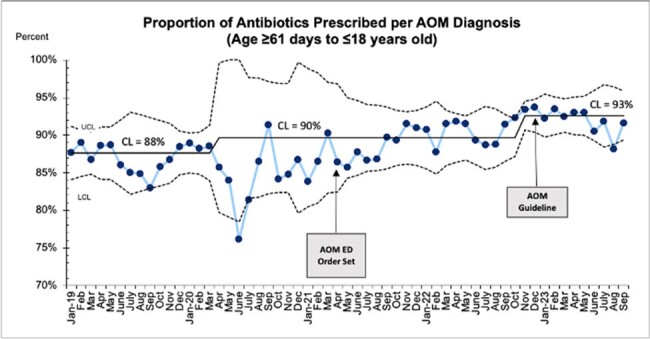
Proportion of antibiotics prescribed per all AOM diagnoses in patients 61 days to 18 years old.

**Methods:**

A bundled intervention for AOM included (1) an electronic health record (EHR) order set that preselected a 5-day antibiotic duration for children 24 months and older (April 2021) and (2) creation of a local clinical care pathway (CCP) for AOM (December 2022). The CCP encouraged observation and pain management in children 24 months and older, as well as children 6-23 months with unilateral, non-severe AOM. A retrospective review of patients 61 days to 18 years old presenting to emergency and urgent care centers between January 2019 through September 2023 was conducted. Outcomes were (1) rates of antibiotic prescribing (enteral or ceftriaxone) in patients 61 days to 18 years old, (2) treatment duration of 5 or fewer days (excluding azithromycin) in patients 24 months to 18 years old, and (3) first-line antibiotic choice (amoxicillin) in patients 61 days to 18 years old. Statistical process control charts with upper and lower control limits and “special cause” variation by standard definitions were used to analyze outcomes.

**Figure 2.**
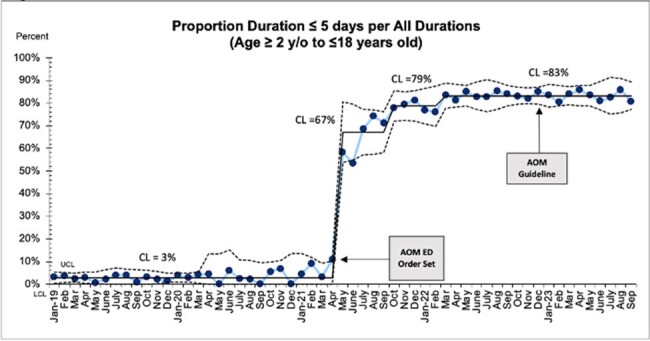
Proportion of patients with duration of 5 or fewer days compared to all duration in patients 2 years to 18 years old.

**Results:**

A total of 34,324 patients were included. Rates of antibiotic prescribing remained high throughout the study (88 to 93%), with special cause variation detected (Figure 1). After EMR order-set development, increased compliance with recommended duration of 5 or fewer days was observed (3% to 83%), with special cause variation throughout the study (Figure 2). Rates of amoxicillin prescribing decreased throughout the study (77 to 74%), with special cause variation detected (Figure 3).

**Figure 3.**
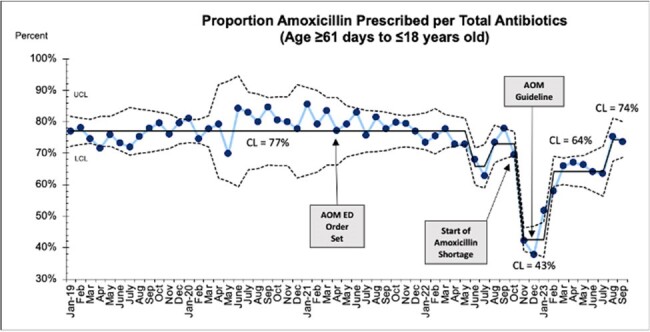
Proportion of amoxicillin prescribed per all antibiotics in patients 61 days to 18 years old.

**Conclusion:**

Implementation of an EHR order set resulted in shorter durations of prescribed antibiotics for AOM in patients older than 24 months. Antibiotic prescribing rates increased, and amoxicillin prescribing decreased throughout study period. The effect of the amoxicillin shortage, which started in October 2022, likely played a role in decreased amoxicillin prescribing rates. This project indicated that further dissemination of the new CCP and de-implementation of previous practices is needed to achieve improvement in antibiotic prescribing and first line antibiotic use.

**Disclosures:**

**All Authors**: No reported disclosures

